# Supplementary figures and images for: Polyamine biosynthesis in Xenopus laevis: the xlAZIN2/xlODC2 gene encodes a lysine/ornithine decarboxylase
Source: PLoS One. 2019 Sep 11;14(9):e0218500. doi: 10.1371/journal.pone.0218500 (PMC6738921; doi:10.1371/journal.pone.0218500)

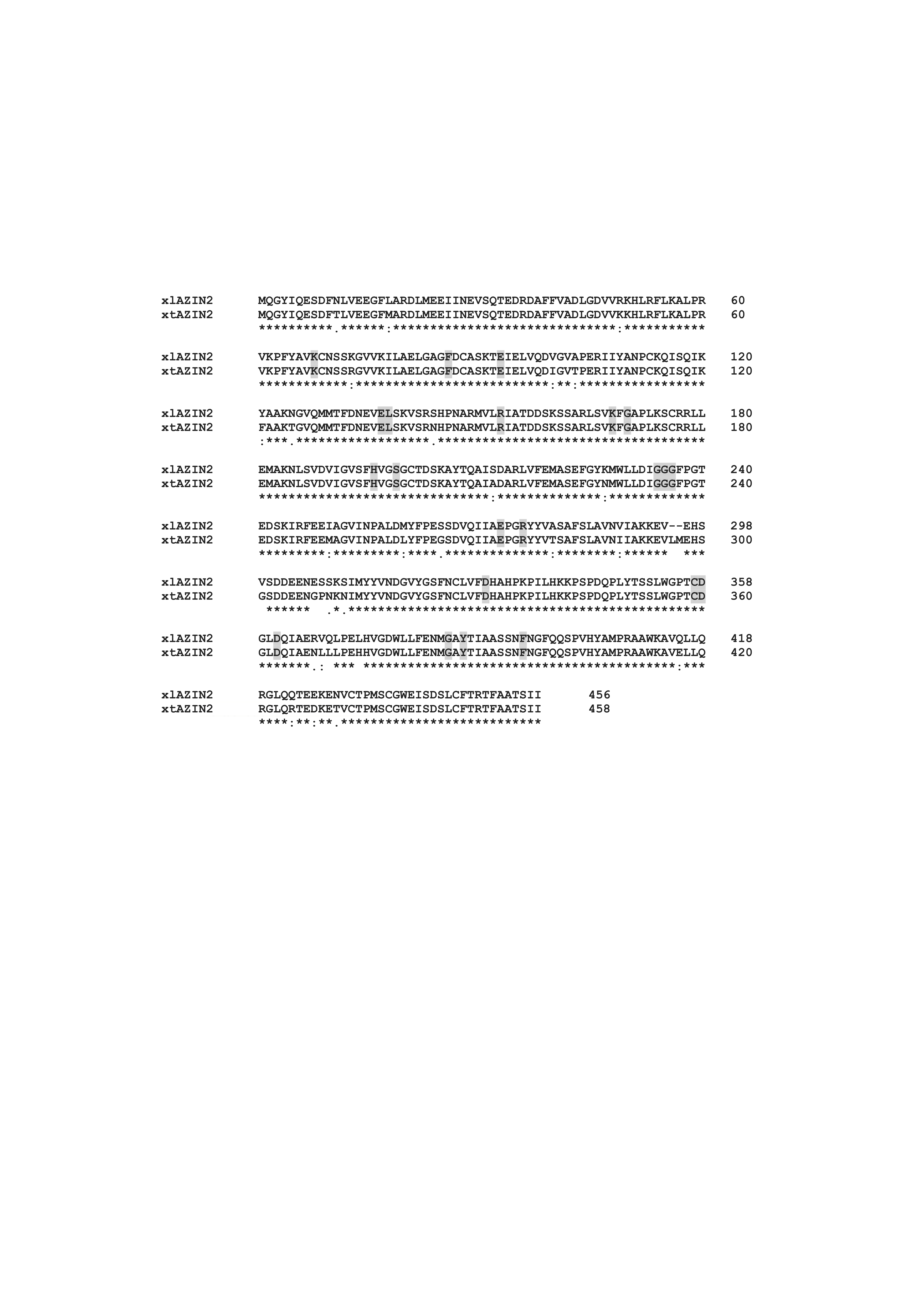

Supplement: S1 Fig — Asterisks represent amino acid identity; colon and dots represent amino acid similarity between the proteins. Grey background indicates amino acid residues associated with the catalytic activity of mODC that are conserved in the Xenopus homologues. (TIFF) [file pone.0218500.s001.tiff]

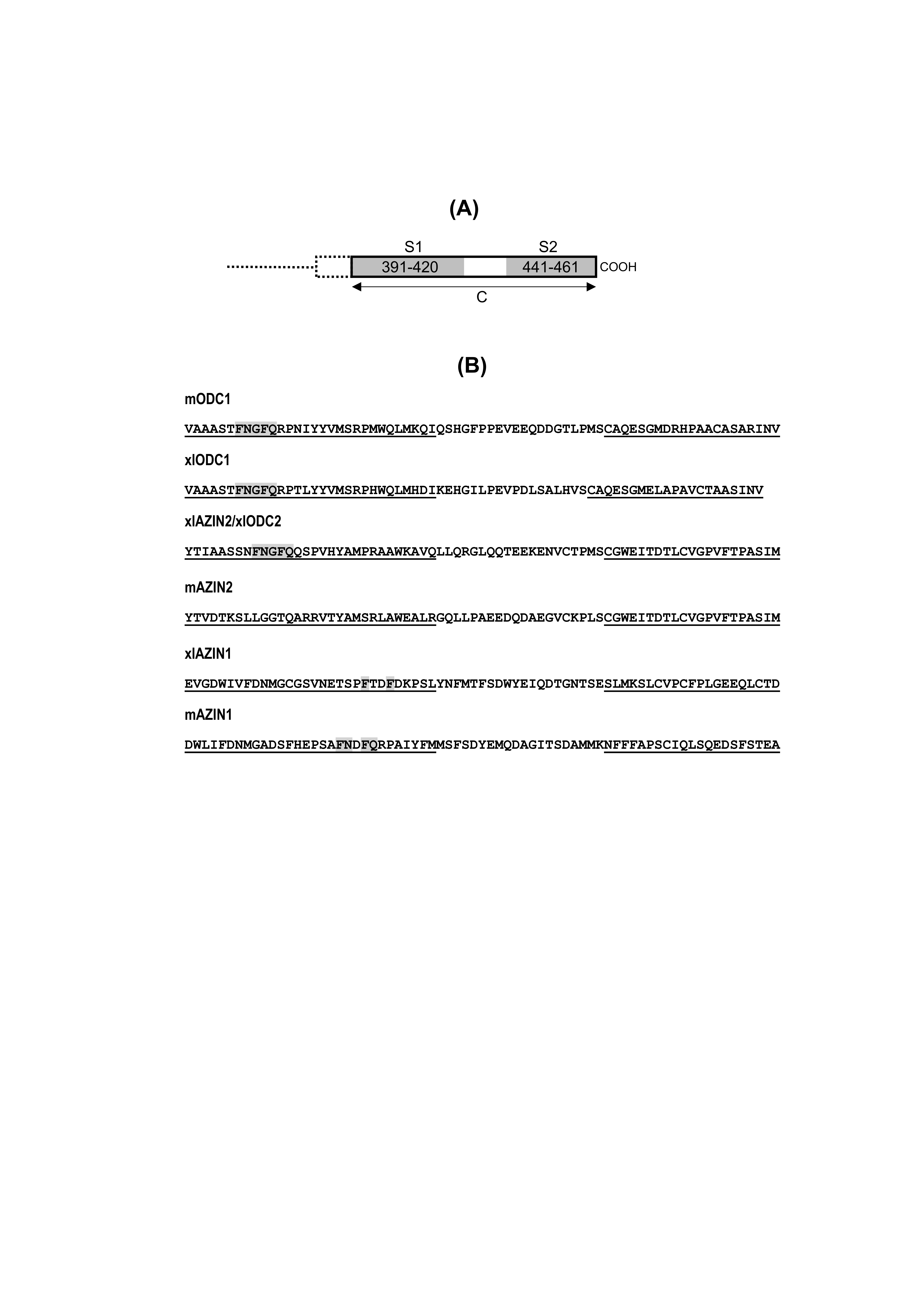

Supplement: S2 Fig — (A) Scheme of the C-terminal region of mODC, where C represents the ~70 amino acid residues, and S1 and S2 the two subregions that may be important for proteasomal degradation of ODC induced by AZ1. (B) Detailed sequence of the C-terminal region of mODC and its different paralogues and orthologues. Sequences corresponding to S1 (residues 391–420) and S2 (residues 441–461) are underlined. (TIFF) [file pone.0218500.s002.tiff]

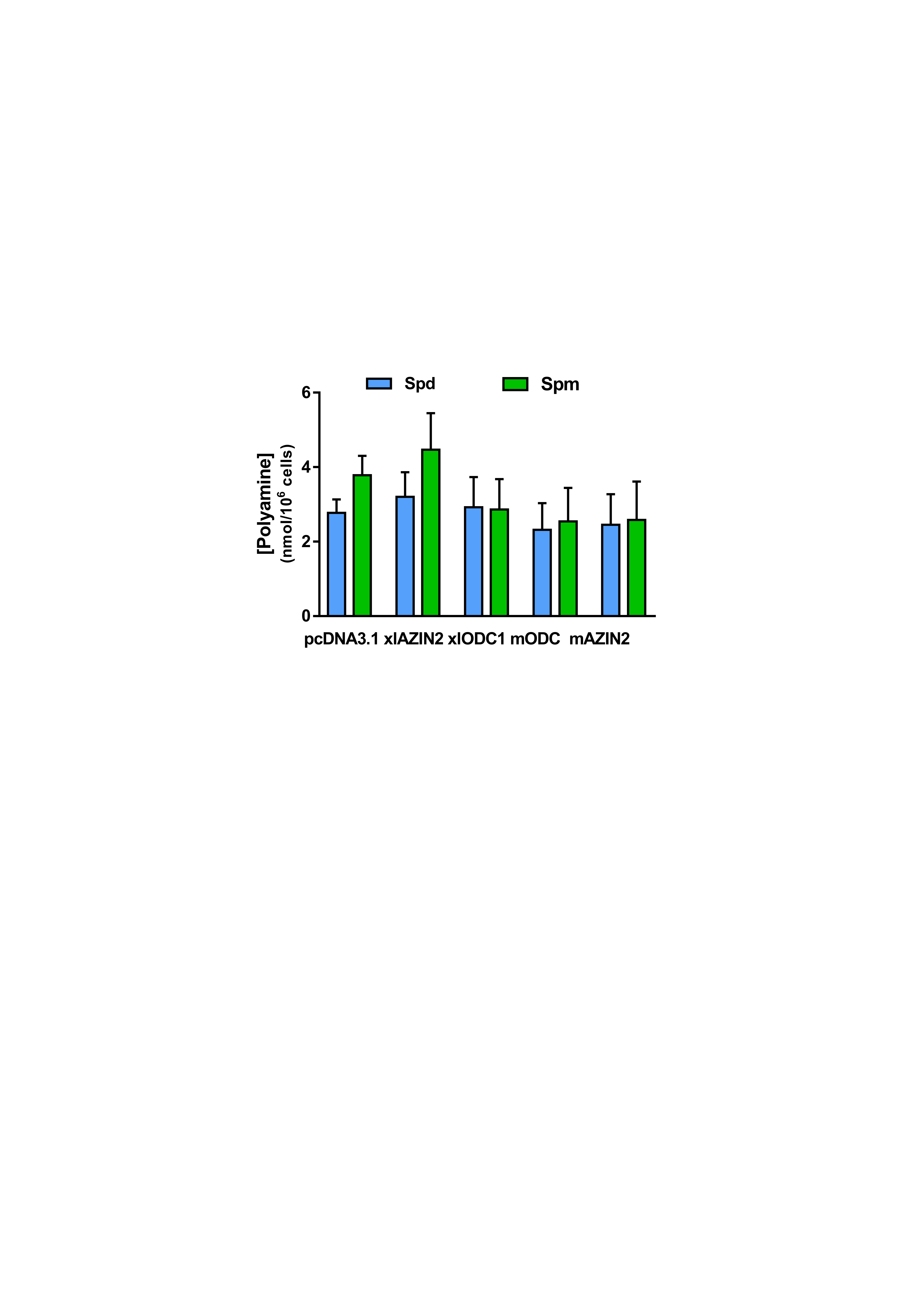

Supplement: S3 Fig — Polyamines were analyzed by HPLC in cell homogenates 16 h after transfection. Neither spermidine (Spd) nor spermine (Spm) were detected in the culture media. (TIF) [file pone.0218500.s003.tif]

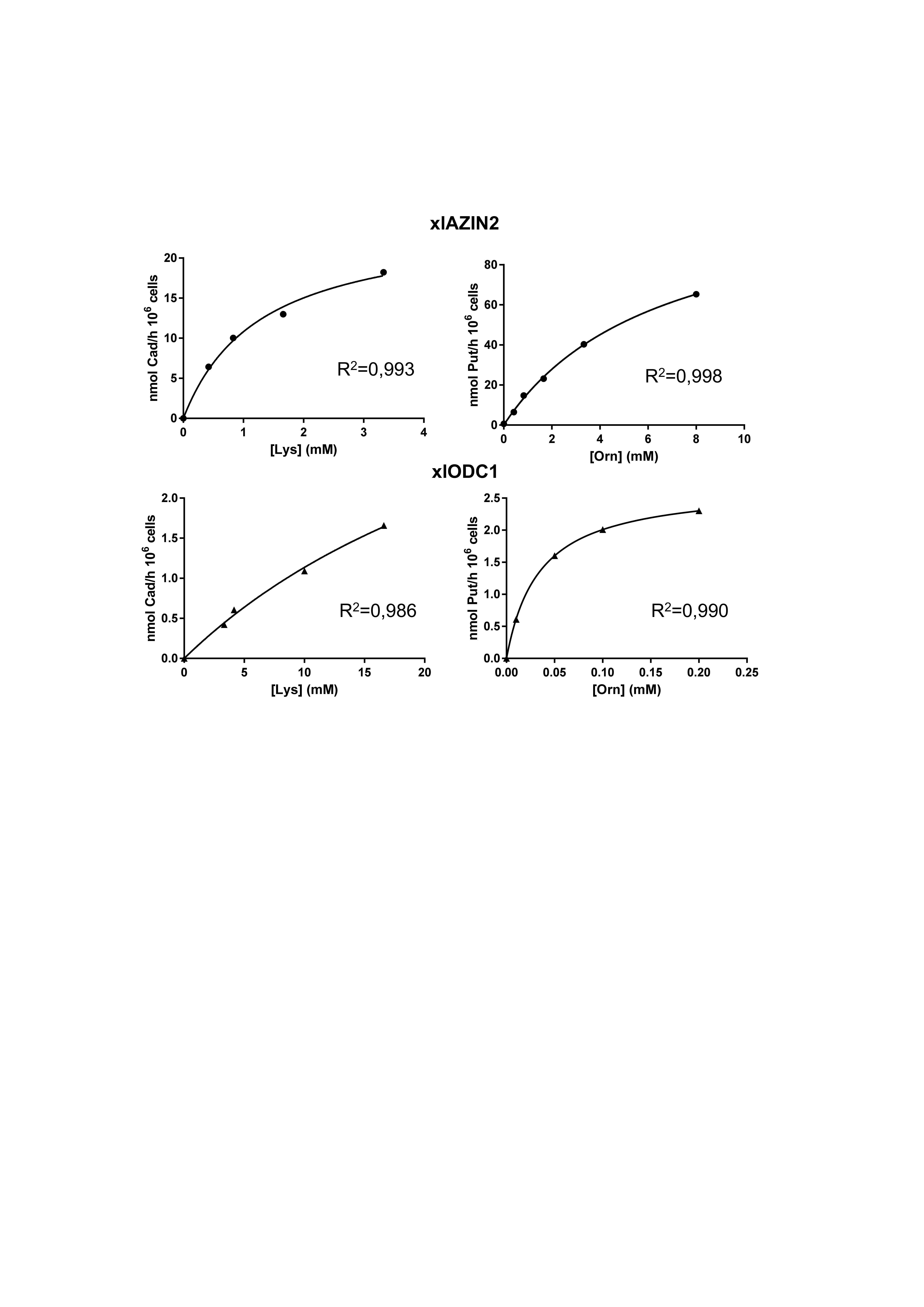

Supplement: S4 Fig — The GraphPad Prism calculator was used and correlation coefficient (R2) for each curve are given. Values of Km and Vm are shown in Table 2. (TIF) [file pone.0218500.s004.tif]

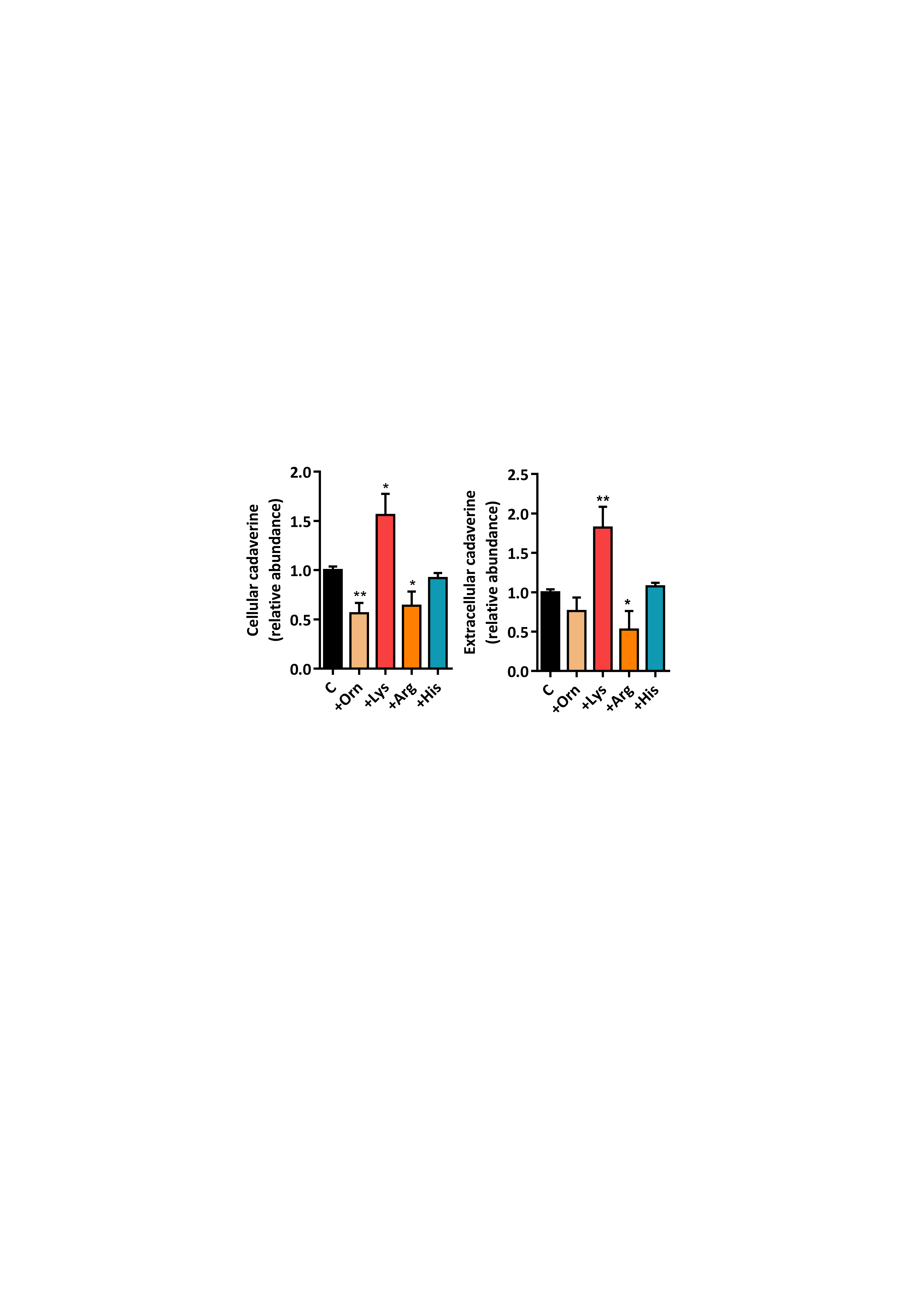

Supplement: S5 Fig — Transfected cells were supplemented with 1 mM L-lysine, L-ornithine, L-arginine or L-histidine. Amino acid concentration in DMEM non-supplemented media: 0.797 mM L-lysine, 0.389 mM L-arginine and 0.20 mM L-histidine. (*) P<0.05 vs control; (**) P<0.01 vs control. C: control (non-supplemented media). (TIF) [file pone.0218500.s005.tif]

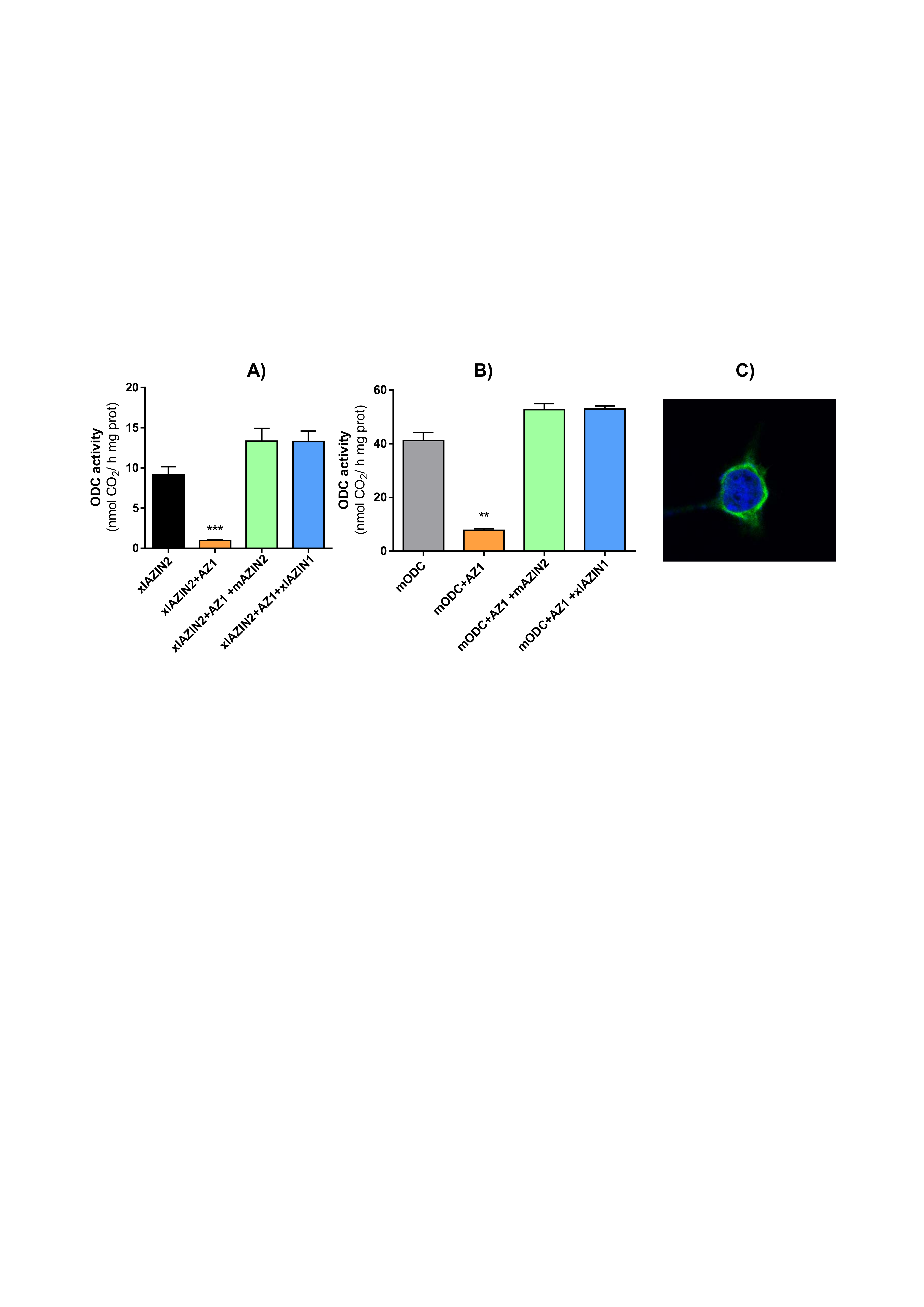

Supplement: S6 Fig — HEK293T cells were transfected with different constructs and ODC activity was measured in cell homogenates 16 h after transfection. The amount of AZ1 plasmid in the transfection assays was 1/10 of the other plasmids. xlAZIN1 showed a cytosolic location, similar to that of xlAZIN2 or mODC shown in Fig 7. (***) P<0.001 vs the other columns; (**) P<0.01 vs the other columns. (TIF) [file pone.0218500.s006.tif]
