# Supplementary material for: Polyamine biosynthesis in Xenopus laevis: the xlAZIN2/xlODC2 gene encodes a lysine/ornithine decarboxylase
Source: PLoS One. 2019 Sep 11;14(9):e0218500. doi: 10.1371/journal.pone.0218500 (PMC6738921; doi:10.1371/journal.pone.0218500)
Supplement: S1 Table — C: terminal region from residues 391 to 461 in mODC; S1 and S2 are two subregions that may be important for ODC proteasomal degradation induced by AZ1; S1: residues 391–423; S2: residues 441–461. (See S2 Fig). (PDF) [file pone.0218500.s007.pdf]

	xlODC1			xlAZIN2			xlAZIN2mAZIN2			xlAZIN1			mAZIN1			mAZIN2		
mODC	C	S1	S2	C	S1	S2	C	S1	S2	C	S1	S2	C	S1	S2	C	S1	S2
	63	77	66	31	40	14	29	40	9	17	26	9	22	33	19	21	26	9
